# Effectiveness of Nutrition Interventions in Vending Machines to Encourage the Purchase and Consumption of Healthier Food and Drinks in the University Setting: A Systematic Review

**DOI:** 10.3390/nu12030876

**Published:** 2020-03-24

**Authors:** Megan C. Whatnall, Amanda J. Patterson, Melinda J. Hutchesson

**Affiliations:** 1 School of Health Sciences, Faculty of Health and Medicine, University of Newcastle, Callaghan 2308, Australia; megan.whatnall@newcastle.edu.au (M.C.W.); amanda.patterson@newcastle.edu.au (A.J.P.); 2 Priority Research Centre for Physical Activity and Nutrition, University of Newcastle, Callaghan 2308, Australia

**Keywords:** nutrition intervention, vending machine, food dispensers, university, college

## Abstract

University food environments typically offer an abundance of unhealthy foods, including through vending machines. This review evaluated the effectiveness of nutrition interventions in vending machines in the university setting. Ten databases were searched for experimental studies published up to July 2019, evaluating nutrition interventions that aimed to encourage the purchase or consumption of healthier foods and drinks in vending machines in the university setting. In total, 401 articles were identified, and 13 studies were included. Studies were pre-post test (*n* = 7, 54%), randomized controlled trials (RCTs) (*n* = 5, 38%), and non-randomized controlled trial (*n* = 1, 8%). Most studies were from the USA (*n* = 10, 77%) and were published between 2014 and 2018 (*n* = 9, 69%). Eight interventions (62%) reported positive change in outcomes, including increased number/proportion of sales or revenue from healthier items (*n* = 6), improved adherence to guidelines for the ratio of healthy/unhealthy products available (*n* = 1), and improved consumer perception of items available (*n* = 1). Effective interventions involved the promotion, reduced pricing, increased availability, and/or optimized product placement of healthier items within vending machines. Strategies to improve the nutritional quality of food and drinks in vending machines are warranted. This may be achieved by making healthier options more available and promoting them; however, more robust intervention studies are needed to determine effectiveness.

## 1. Introduction

University food environments typically offer a high proportion of unhealthy food and beverage options. For example, in an audit of 252 food and beverage outlets across seven Australian institutions in 2014, two-thirds of the available items were sugar-sweetened beverages, chocolate, high energy per serve (>600 kJ) foods, chips, or confectionary [1]. Further, an audit of 15 USA institutions, including 263 dining outlets, found that less than half (40%) offered healthy main dishes [2]. This is an issue, as globally, universities are a place of work and study for millions of individuals [3,4]. Therefore, university food environments are potentially contributing to poor dietary intakes, as well as the associated overweight/obesity risk, among a large sector of the population [5,6]. Further, there is an international movement toward Health Promoting Universities and Colleges in accordance with the 2015 Okanagan Charter [7]. Current and evidence-based strategies are needed to address the calls to action of the Okanagan Charter, which include to “Embed health into all aspects of campus culture…” and “Lead health promotion action and collaboration locally and globally” [7]. Evidently, intervention strategies are needed to improve university food environments. 

Vending machines are one component of the university food environment where the availability of unhealthy items is especially high. In vending machine audit studies from universities in Australia, UK, and USA, the proportion of unhealthy items available ranged from 85% to 100% for foods and from 49% to 80% for beverages [8,9,10]. Intervention strategies to improve the nutritional quality of items available in vending machines have demonstrated some success [11,12]. In 2015, Grech et al. conducted a systematic review of nutrition intervention studies in vending machines in all populations/settings, with five of 12 included studies conducted in the university setting [12]. Of the 12 included studies, eight reported significant findings following intervention, with the most effective strategies being price reductions and increasing the availability of healthier items, resulting in increases in sales for healthier items. As four years has elapsed since this review was conducted and relevant studies in the university setting have since been published [13,14], an updated review of the evidence is warranted. 

Therefore, the aim of this systematic review was to evaluate the current evidence examining the effectiveness of nutrition interventions in vending machines to encourage the purchase and consumption of healthier food and drinks in the university setting. 

## 2. Materials and Methods 

The review protocol was registered with PROSPERO (CRD42019141638) and adheres to Preferred Reporting Items for Systematic Reviews and Meta-Analyses (PRISMA) guidelines [15]. 

### 2.1. Criteria for Study Inclusion 

#### 2.1.1. Participants/Population

Studies undertaken solely in the University setting were included. For studies that assessed participant-related outcomes, participants had to be university staff and/or students.

#### 2.1.2. Intervention 

All nutrition interventions in vending machines that aimed to encourage the purchase and consumption of healthier food and drinks were included (i.e., increasing the availability of healthier food and drinks within vending machines or modifying portion size, promotion of healthier food and drink choices (e.g., signage), product placement within vending machines, or price alterations).

#### 2.1.3. Comparator

For studies with comparison groups, the comparison was a no-intervention control group, or another type of nutritional intervention.

#### 2.1.4. Outcomes

Outcomes of interest included, purchase/sales of food and drinks from vending machines, dietary behavior change, change in food/drinks available within vending machines, and the acceptability of/satisfaction with the vending machine intervention. 

#### 2.1.5. Study Design

All types of experimental studies (i.e., randomized controlled trials (RCTs), pseudo-RCTs, pre-post studies) were included. 

### 2.2. Search Strategy

Ten electronic databases were searched using predetermined keywords and index terms, including CENTRAL, CINAHL, Cochrane Reviews, EMBASE, EMCARE, ERIC, MEDLINE, PsycINFO, Scopus, and Web of Science. Searches were limited to studies published in the English language up to 23 July 2019 (Supplementary Table S1). The reference lists of all included papers were also searched to identify any additional papers. 

### 2.3. Selection of Studies

All studies identified during the database and reference list searches were assessed for relevance to the review based on the information contained in the title, abstract, and description/Medical Subject (MESH) heading by two independent reviewers (M.J.H. and M.C.W.). For all studies that met the inclusion criteria, or if this was unclear, the full article was retrieved. Papers selected for retrieval were assessed by two independent reviewers to determine inclusion (M.J.H. and M.C.W.). In case of disagreement, a third independent reviewer made the final decision (A.J.P.). For studies deemed ineligible for inclusion in the review, the reasons for exclusion were recorded. 

### 2.4. Data Extraction

Data were extracted by one reviewer (M.C.W., C.Y.L., or H.S.N.) and checked by a second reviewer (M.C.W. or M.J.H.) using a standardized data extraction tool developed by the authors. The following information was extracted: study characteristics (authors, date of publication, study design, and study setting/population); description of intervention and comparison groups; and description of study outcomes (e.g., outcome, measurement tool, findings). 

### 2.5. Risk of Bias/Quality Assessment

Risk of bias was assessed by two independent reviewers (M.C.W., C.Y.L., H.S.N., or M.J.H.), with a third independent reviewer making the final decision in the case of disagreement (A.P.). The Academy of Nutrition and Dietetics Quality Criteria Checklist for Primary Research was used to assess risk of bias [16]. This tool assesses 10 criteria relating to (1) clarity of the research question, (2) bias in the selection of study participants/subjects, (3) comparability of study groups, (4) whether methods of handling withdrawals were described, (5) the use of blinding, (6) whether intervention and comparators were described in detail, (7) whether outcomes were defined clearly and measurements were valid and reliable, (8) appropriateness of statistical analyses, (9) whether conclusions are supported by results and consider biases and limitations, and (10) whether study funding or conflicts of interest are likely to have introduced bias. Each of the criterion are rated as yes/low risk of bias, no/high risk of bias or unclear, from which the overall study quality is rated as positive/low risk of bias (if criteria 2, 3, 6, 7, and one other are yes), negative/high risk of bias (if six or more criteria are no), or neutral (if criteria 2, 3, 6, and/or 7 are no, unclear, or not applicable). 

### 2.6. Data Synthesis 

Results are described in narrative form. Studies are grouped by type of intervention strategies used, i.e., increasing the availability of healthier food and drinks within vending machines, promotion of healthier choices, product placement within vending machines, or price alterations. Due to the heterogeneity of study outcomes, meta-analysis was not conducted. 

## 3. Results

### 3.1. Description of Included Studies

**Figure 1:**
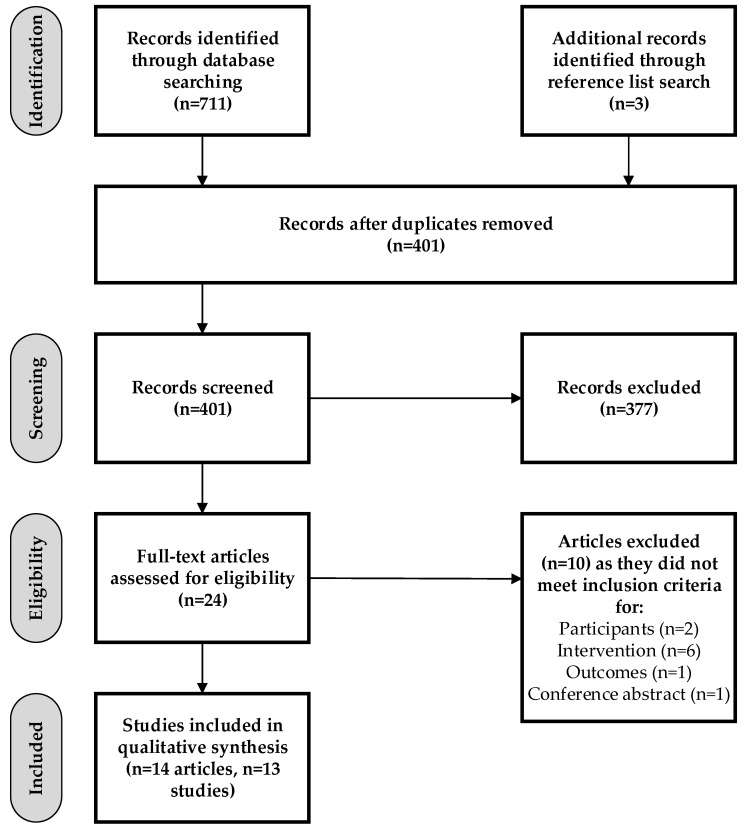
Flow diagram of included studies.

Of the 401 articles identified, 14 articles reporting on 13 studies met the inclusion criteria (Figure 1). Seven studies were of pre-post test design (54%), five were RCTs (38%), and one was a non-randomized controlled trial (8%) (Table 1). The majority of studies were conducted in the USA (*n* = 10, 77%), with one study each conducted in Australia, Singapore, and Italy. Studies were published between 1978 and 2018, with the majority published between 2014 and 2018 (*n* = 9, 69%). All studies were conducted at a single university. The mean number of vending machines included in interventions was 22 (range 2–97); however, in two studies the number of vending machines was not reported. In 12 studies, interventions included a sample of the vending machines available on campus, while one study included all vending machines on campus. In six studies, the interventions targeted food items only, three studies targeted drink items only, three studies targeted both food and drink items, and in one study this was unclear. The types of intervention strategies included promotion of healthier food and drink choices (*n* = 11, 85%) [13,14,17,18,19,20,21,22,23,24,25], increasing the availability of healthier food and drinks within vending machines (*n* = 8, 62%) [13,14,22,23,24,25,26,27], price alterations (*n* = 4, 31%) [13,14,20,24], and modifying product placement within vending machines (*n* = 2, 15%) [14,23]. Interventions in seven studies involved more than one of these strategies, with three studies using two strategies [20,22,25], three studies using three strategies [13,23,24], and one study using four strategies [14]. The most common combinations of strategies were increasing the availability and promotion of healthier food and/or drink choices [22,25] and increasing the availability and promotion of healthier food and/or drink choices and price alterations [13,24], used in two studies each. The outcome measures included purchases/sales from vending machines in all except one study (*n* = 12, 92%) [13,14,17,18,19,20,21,22,23,24,25,26,28] (Table 2). Two studies (15%) also measured the acceptability of/satisfaction with the intervention [22,26], and one study (8%) also measured dietary behavior change [22]. The outcome measure of the remaining study was the change in food/drinks available within vending machines [27].

### 3.2. Interventions Involving the Promotion of Healthier Food and Drink Choices 

In four studies (2 RCTs, 2 pre-post tests), interventions involved the promotion of healthier food and/or drink choices as the sole intervention strategy [17,18,19,21]. Of these, two studies (50%) reported positive change in outcomes [17,21]. Bergen et al. reported a 25% increase in revenue, of which 70% came from healthier drink choices, during a five-week RCT where water and zero-energy soft drinks were promoted via ‘0 Calorie, 0 Carb’ labels and motivational/educational posters [17]. Larson-Brown et al. reported a 14% increase in monthly sales and a 4% increase in the proportion of sales from more nutritious foods, during a four-week intervention where more nutritious foods were promoted by the addition of nutrition information cards for all items in vending machines [21]. In the pre-post test study by Brown et al. and the RCT by Dingman et al., healthier food and drink choices were promoted by a traffic light color coding system or nutrition information panels on vending machines, respectively, as well as additional advertising of the interventions (via posters on campus or emails to resident students) [18,19]. Although Brown et al. reported increases in the sale of healthier (green) items and Dingman et al. reported increases in the proportion of healthier items sold, in both studies the increases were not significant.

### 3.3. Interventions Involving Increasing the Availability of Healthier Food and Drink Choices 

In two studies (both pre-post test), interventions involved increasing the availability of healthier food and/or drink choices as the sole intervention strategy [26,27], with one reporting positive change in outcomes and the other reporting mixed findings. In the study by Tsai et al., the ratio of healthy to unhealthy items available across all vending machines on campus changed from 23:77 to 77:23, and availability of sugar-sweetened drinks from 56% of drinks to 0%, 12 months after the university-wide implementation of a healthy food and drink framework based on the Australian Dietary Guidelines [27]. In the study by Lapp et al., 45% of vending machine items were replaced with healthier choices over a two-week intervention period [26]. For example, swapping candies for dried fruit. Consumer (student) perceptions, measured by self-report survey, significantly changed in favor of perceiving the options as healthier and more likely to sustain them through university classes, however vending machine sales did not change.

### 3.4. Interventions Involving Multiple Strategies 

In seven studies (3 RCTs, 3 pre-post tests, 1 non-randomized controlled trial), interventions involved a combination of two or more strategies [13,14,20,22,23,24,25]. Of these, four studies (57%) reported positive change in outcomes [13,14,20,23]. 

In two pre-post test studies, interventions involved increasing the availability and promotion of healthier food and/or drink choices [22,25]. Rose et al. installed new vending machines alongside existing vending machines in two student residence buildings, which sold only low-fat flavored milk and were promoted via flyers. However, there were no significant changes in students’ dietary intake of calcium, measured by a food frequency questionnaire after the two-month intervention [22]. Hoerr et al. involved a two-stage intervention where the proportion of healthier items available was first increased from 12% to 38%, followed by the addition of nutrition information cards for all items [25]. While the proportion of healthier items sold increased with each stage, these changes were not statistically significant. The overall machine sales significantly declined after stage one, and significantly increased after stage two; however, they were still lower than baseline sales.

In two RCT studies, interventions involved increasing the availability and promotion of healthier food and/or drink choices and price alterations [13,24]. The study by Hua et al. involved a 2 × 2 × 2 factorial design, with factors including increased availability of healthier products (100% of snack/75% of beverage options meeting healthy guidelines), price reductions (25% price reduction on snack items meeting healthy guidelines/$1 water) and promotional signage (promoting nutrition information of items meeting healthy guidelines and/or price reductions) [13]. Results were mixed: for beverage vending machines, there were significant increases in sales for machines offering healthier products with signage promoting these products, both with and without price reductions; for snack machines, those offering healthier products with signage promoting these products had increased revenue, but all other combinations resulted in significantly decreased sales and revenue. Seah et al. implemented the same changes in availability and price of beverages across all groups; 44% of available beverages were lower-sugar options, and a 10% lower price was put on these than on higher-sugar options. Therefore the availability and price of beverages was standardized, with the component of difference being the way these changes were framed in intervention groups (tax on high-sugar beverages or subsidy for lower-sugar beverages) versus no promotion messages for the control group [24]. No significant between group differences in sales were found over the nine-week intervention. 

One study involved the promotion of healthier food and/or drink choices and price alterations. In the pre-post test study by French et al., the intervention involved a 50% price reduction on low-fat snack items with promotion via bright signage in and on machines and reported an increase from 25.7% of total sales at baseline to 45.8% of sales during the three-week intervention (i.e., a 20.1% increase) [20]. 

One study involved increasing the availability and promotion of healthier food and/or drink choices and modifying product placement. The RCT by Rosi et al. included a two-stage intervention trial where the availability of items and product placement were changed first (healthy:unhealthy items 50% each and products arranged healthiest to least healthy from left to right) [23]. Stage two involved the addition of nutrition information in, and next to, a subset of machines in the form of either nutrition content and claims or a star rating system to indicate healthiness of products. Following stage one, the proportion of sales from healthier items increased by 32% (*p* < 0.001) with no significant change in total sales, while after stage two the proportion of least healthy items sold was significantly lower in the machines displaying star ratings than those with no nutrition information (19% vs. 28%, *p* < 0.05). 

One study, the non-randomized controlled trial by Viana et al., involved all four strategies, (increasing the availability and promotion of healthier food and/or drink choices, modifying product placement, and price alterations). In this study, healthier items were made more available (minimum 25% of options), optimally placed within vending machines (grouped together including dedicated rows placed at eye level) and promoted via ‘Eat Well’ stickers, while the price of candy bars was increased by 25% [14]. During the two-month intervention, the proportion of healthier items sold was 21.3% in intervention compared with 1.3% in control machines (i.e., a difference of 20.0%) with no significant difference in revenue. As a secondary measure, point-of-purchase surveys were conducted to compare consumers intended and actual purchases, finding that 63% of customers had no clear purchase intention, and of these, customers were more likely to purchase healthier items when using intervention versus control machines (50% vs. 10%, *p* < 0.01). 

### 3.5. Risk-of-Bias of Included Studies

The risk-of-bias assessment is summarized in Table 3. Seven studies (54%) were rated as positive quality i.e. low risk of bias, and the remaining six studies (46%) were rated as neutral. The studies rated neutral quality tended to lack detail in the reporting of the selection of study participants/subjects and outcome measures used, and therefore there was an unclear risk of bias for these criterion.

## 4. Discussion

This systematic review evaluated the effectiveness of nutrition interventions in vending machines in the university setting. Thirteen studies were included, of which seven were pre-post test, five were RCTs, and one was a non-randomized controlled trial. Just over half of the studies reported positive findings (*n* = 8, 62%), predominantly being increases in the sales or proportion of sales from healthier food or drink items. One study reported negative findings, being a significant reduction in sales and revenue, while four studies reported neutral findings. Intervention strategies included promoting, increasing the availability, or altering the price or placement of healthier food and drinks within vending machines. The most common intervention strategy was the promotion of healthier food and drink choices, used in 11 studies, of which six studies (55%) reported positive findings. The findings provide support for the implementation of nutrition interventions in vending machines in the university setting, as well as a basis for more robust intervention studies in the future. 

In this review, interventions involving the promotion of healthier food and drink choices were effective in six of 11 studies, while those involving increasing the availability of healthier choices were effective in five of eight studies. Interventions involving altering the price and placement of healthier food and drinks were effective in three of four, and two of two studies, respectively. In the majority of these studies, the effective outcome was an increase in sales from healthier items. There is some consistency in these findings compared with the 2015 review of vending machine interventions in all populations and settings by Grech et al. [12]. The most effective strategies in the Grech et al. review were price reductions and increasing the availability of healthier items, with the effective outcome being an increase in sales from healthier items. However, in both reviews, it is difficult to determine the most effective intervention strategies as they were predominantly used in combination and due to the small number of included studies and heterogeneity in outcome measurements. Further, as the majority of studies were pre-post test design, few studies have compared individual intervention approaches. It may also be that different intervention strategies are effective for foods versus drinks; however, this is unclear from the current review. Additionally, the effect of the interventions on dietary behaviors and their acceptability is unclear, as only a small number of studies have measured these outcomes. Only two studies in this review included an outcome measure of acceptability/satisfaction, and only one study measured dietary intake. Lapp et al. reported a positive shift in student perceptions of items being healthier and more sustaining where the intervention involved an increased availability of healthier items. However, Rose et al. reported no change in dietary calcium intake following the implementation of new milk vending machines alongside existing vending machines and found that three main factors influenced student’s attitudes to milk vending machines (convenience/likeability, family/friend influence, and health/experience). 

Overall, the findings of this review indicate that nutrition interventions in vending machines in the university setting can improve the availability and sales of healthier items. However, as the majority of studies were of pre-post test design, there is a need for more robust intervention studies to determine their effectiveness, including the individual and combined intervention strategies that are most effective. Further, it is unclear whether studies were powered to detect changes in outcomes. Therefore, adequately powered randomized controlled trial studies are needed that compare different intervention strategies and combinations of strategies. Future studies should also include measures of dietary intake/behavior and/or intervention feasibility/acceptability, in addition to sales/revenue [12]. These are important outcomes to measure in terms of developing and implementing effective and sustainable interventions to improve this aspect of the university food environment. Given the changing nature of food environments and labeling policies and guidelines, future studies should also evaluate the impacts of relevant policy or guidelines such as the U.S. Food and Drug Administration Vending Machine Labeling Requirements [29] and, in Australia, the Victorian Government Healthy Choices Framework [30]. In regard to the implications for practice, the review findings are useful to inform the development of future interventions, by providing examples of how the different intervention strategies can be implemented. For example, promoting healthier food and drink choices can be achieved by placing educational and motivational information cards/posters in or on vending machines, and increasing the availability of healthier choices can be achieved by implementing university-wide guidelines on product availability. Further, the finding that nutrition interventions in university vending machines can improve the availability and sales of healthier items should be used by university health promotion providers to advocate for action to improve the university food environment. This action should include university-wide policy change/health promotion initiatives and a consistent approach with other facets of university health promotion.

### 4.1. Strengths and Limitations of the Included Studies

The risk-of-bias assessment identified that just over half of the studies had a low risk of bias overall, while the remaining were neutral. For all studies, there was a clear statement of the research question, intervention and comparators were adequately described, and statistical analyses and conclusions were appropriate. In terms of limitations, there was an unclear risk of bias for some studies in terms of selection of study participants/subjects and outcome measures used, due to a lack of detail reported. Other limitations to the validity and generalizability of study findings include the small number of included studies, that the majority of the included studies were pre-post test design without a control group and were conducted in the USA, and that few studies assessed outcomes in the long term. Study outcomes were primarily purchase/sales from vending machines, with only three studies including outcomes of dietary intake or acceptability (i.e., actual behavior change or feasibility). Therefore, further study of the impact of interventions on these other outcomes is needed. 

### 4.2. Strengths and Limitations of the Review 

The main strengths of the review include the comprehensive search and screening strategies used for the identification of relevant studies and the use of a reliable tool to assess risk of bias. Restricting studies to those published in the English language is a limitation, in terms of potentially excluding relevant studies and the generalizability of the review findings. A further limitation is that food and drinks were considered as healthier as per the definitions and criteria of the individual studies. The heterogeneity in these definitions is a limiting factor in terms of comparing and summarizing study findings [31], however, it is also because of this heterogeneity that an overarching definition could not be applied. 

## 5. Conclusions

Strategies to improve the nutritional quality of food and drinks in vending machines in the university setting are warranted. This review identified that promoting, increasing the availability, and altering the price and placement of healthier food and drinks within vending machines may positively increase sales or proportion of sales from healthier items. However, further evidence from more robust intervention studies is needed to determine the most effective strategies to improve this aspect of the university food environment, including comparison of individual and combined intervention strategies.

## Figures and Tables

**Table 1:** Nutrition interventions in vending machines in the university setting: study characteristics.

First Author, Year, Country	Study Design; Population/Setting	Intervention Description	Comparator Description	Intervention Type ^a^	Comparator Type ^a^	Intervention Duration
Bergen, 2006, USA [17]	RCT; 1 university, 8 VM	Intervention 1: "0 Calorie, 0 Carbs" labels displayed on selection panels for water and zero-energy soft drinks (*n* = 3). Intervention 2: same labels plus motivational/educational posters encouraging water and zero-energy soft drinks (*n* = 3).	Control: No intervention (*n* = 2)	Promotion	NA	5 weeks
Brown, 2014, USA [18]	Pre-post test; 1 university, 5 VM	Color-coded stickers placed on items (red, yellow, green) to indicate health rating, with a larger sticker on the front of machines explaining the color-coding and matching nutrition information. Posters around campus encouraging more green items, moderate consumption of yellow items, and less red items.	NA	Promotion	NA	2 weeks
Dingman, 2015, USA [19]	RCT; 1 university, 18 VM in student residence halls	Posters next to machines displaying nutrition information panel for each item. Five ’Better Choice’/healthier products promoted on posters and stickers in machines. Residents were also emailed regarding intervention (*n* = 9 machines).	Control: No intervention (*n* = 9 machines)	Promotion	NA	4 weeks
French, 1997, USA [20]	Pre-post test; 1 university, 9 VM	Fifty percent price reduction for low-fat snack items. Bright orange price labels were placed beneath items in the machine, and a bright orange sign on the front of the machine defined low-fat snacks (<3 grams fat per package).	NA	Promotion; Price	NA	3 weeks
Hoerr, 1993, USA [25]	Pre-post test; 1 university, 4 VM	Proportion of healthier/higher Index of Nutritional Quality (INQ) items available increased relative to moderate- and low-INQ items in year 2. In the third year, nutrition information cards were added next to each item in the machines.	NA	Availability; Promotion	NA	3 years
Hua, 2017, USA [13]	RCT; 1 university, 56 VM	2 × 2 × 2 factorial design—8 intervention conditions. Healthier product guidelines (100% food items and 75% beverages meeting healthier guidelines) and/or price changes (25% off healthier products and $1 water) and/or promotional signs (promoting healthier items and/or price reduction).		Availability and/or Price and/or Promotion	Availability and/or Price and/or Promotion	5 months
Lapp, 2014, USA [26]	Pre-post test; 1 university, 2 VM/197 students (77% female, 28% live off-campus)	45% of items in vending machines replaced with healthier choices (criteria based on US Dietary Guidelines).	NA	Availability	NA	2 weeks
Larson-Brown, 1978, USA [21]	Pre-post test; 1 university, number of VM not reported	Nutrition information cards placed next to each item in vending machines (graphs displaying % of dietary recommendations met for selected macro/micronutrients).	NA	Promotion	NA	1 month
Rose, 2018, USA [22]	Pre-post test; 1 university, VM in 2 student dorms (N not reported)/124 students (60% female, 67% freshman)	New vending machines selling only low-fat/fat-free flavored milk installed in two dorm residences, with flyers posted to promote the new machines.	NA	Availability; Promotion	NA	2 months
Rosi, 2017, Italy [23]	Randomized, crossover, controlled study; 1 university, 3 VM	Intervention 2a: Same as active control, plus nutritional content, and claims information provided, alongside products inside and on a digital screen attached to the vending machine. Intervention 2b: Same as active control, plus a star rating of healthiness provided alongside products inside and on a digital screen attached to the vending machine.	Intervention 1 (active control): Proportion of healthy/unhealthy items changed to 50:50 and product placement to healthier to least healthy/left to right.	Availability; Placement; Promotion	Availability; Placement	24 weeks
Seah, 2018, Singapore [24]	Randomized, crossover, controlled study; 1 university, 21 beverage VM	Intervention 1: Tax messages: price change promoted as tax for high-sugar beverages, messages displayed on banners, posters and bright yellow stickers. Intervention 2: Subsidy messages: price change promoted as subsidy for lower-sugar beverages, messages displayed on banners, posters, and bright yellow stickers.	Control: No message	Promotion; Price; Availability	Price; Availability	9 weeks (3 weeks per intervention sequence)
		All groups: Beverage availability (44% lower-sugar options) and prices (10% reduction on lower-sugar options) were standardized across machines.				
Tsai, 2018, Australia [27]	Pre-post test; 1 university, 23 VM	Change in product availability in line with university implementation of the New South Wales Healthy Food and Drink for Health Facilities Framework (75% core items, 25% discretionary items, and 0% sugar-sweetened beverages).	NA	Availability	NA	Ongoing
Viana, 2018, USA [14]	Non-randomized controlled trial; 1 university, 97 VM/100 staff/students (83% students, 53% female, mean age 20 years)	Intervention machines branded with Healthy Campus Initiative stickers including a web address for further info on the intervention and nutrition criteria for healthier products. "Eat Well" stickers to identify healthier products in machines, healthier products accounted for minimum 25% of options within large/small snack categories, and product placement was re-organized for more optimal placement of healthier products (*n* = 36).	No intervention (*n* = 61)		Price	2 months
		All machines: price increase on candy bars from $1 to $1.25.				

^a^ Promotion: promotion of healthier food and/or drink choices; Price: price alterations; Availability: increasing the availability of healthier food and drinks within vending machines; Placement: modifying product placement within vending machines. VM: vending machines.

**Table 2:** Nutrition interventions in vending machines in the university setting: study results.

First Author, Year, Country	Measurement Timepoints	Outcome Measures	Description of Main Findings
Bergen, 2006, USA [17]	Baseline, during, and post (2 weeks data collection baseline and post)	Beverage sales per week; machine revenue per week	Sales: growth of soft drink sales significantly less for intervention 2 vs. control during intervention. No other significant between group differences. Total revenue: increased by 25% during intervention (70.5% of increase from zero-energy soft drinks and water)
Brown, 2014, USA [18]	Baseline and during intervention (2 weeks data collection for each)	Machine sales per 2 weeks period	Sales: decrease in red (−4.84%) and yellow (−15.21%) sticker items and increase in green items (+50.76%). Not significant.
Dingman, 2015, USA [19]	Baseline and during intervention (4 weeks data collection each)	Average calories per snack sold; proportion of ‘Better Choice’ snacks sold	No significant changes in outcomes.
French, 1997, USA [20]	Baseline (4 weeks data collection), during (3 weeks) and post intervention (3 weeks)	Percentage of low-fat snacks purchased; Total number of snacks purchased	Percentage low-fat snacks purchased: significantly increased during (26%–46%, *p* < 0.002) and decreased post intervention (46%–23%, *p* < 0.01). Total number snacks purchased: No significant change.
Hoerr, 1993, USA [25]	Year 1 (Baseline), Year 2, and Year 3 (Intervention) (12 weeks sales data collected/year)	Number/proportion of items sold by INQ category; annual sales	Total sales: significantly decreased in Year 2 (85.7% of Year 1 sales) and significantly increased in Year 3 (92.5% of Year 1 sales). Proportion of sales for high-INQ items: increased (Year 1: 9%, Year 2: 26%, Year 3: 27%), however not significant.
Hua, 2017, USA [13]	Baseline and during intervention (5 months data collection in 2014/2015)	Total food/beverage units sold; Machine revenue	Snack machines—Healthier products available + promotions = increase in revenue (+$1039, *p* < 0.05); only healthier products available + price reduction = decline in units sold (-448 units, *p* < 0.05) and revenue (−$1287.33, *p* < 0.05); machines met healthier product guidelines + promotions (both with/without price changes) = decrease in revenue (*p* < 0.05); machines met product guidelines = decrease in revenue (*p* < 0.05). Beverage machines—Machines met healthier product guidelines = increased units sold; machines met healthier product guidelines, price change, + promotions = increased units sold (+66, *p* < 0.005); machines met healthier guidelines + promotions = increased units sold (+204, *p* < 0.05).
Lapp, 2014, USA [26]	Baseline and post intervention	Self-report survey: 1) Perceptions and 2) Usefulness of items available (scale 1–10); and 3) Frequency of purchase	Perceptions: foods perceived as significantly more healthy at post test (+0.4/10, *p* < 0.05), and significantly more useful to help students get through class (+0.5/10, *p* < 0.05). Purchasing: significantly declined pre to post overall (69% vs. 57% purchased in previous week, *p* = 0.01) and for the 2 intervention machines but not significantly (29% vs. 26%, *p* = 0.05).
Larson-Brown, 1978, USA [21]	Baseline and during intervention (1-month data collection each)	Monthly machine sales; % sales per more/less nutritious foods (overall and by food category)	Monthly sales: increased (26,558–30,371 units). Proportion of sales of more nutritious foods: significantly increased (49.8%–53.7%).
Rose, 2018, USA [22]	Baseline and post intervention (surveys) and during intervention (machine sales data)	Objective data: Machine sales/month. Self-report survey: (1) Calcium intake and milk servings/day (food frequency questionnaire), (2) Attitudes (e.g., convenience, scale 1–5)	Sales: 98–159 bottles sold/$171.50–$278.25 per month during intervention. Calcium intake: No significant changes. Attitudes concerning milk vending: three factors identified—convenience/likeability, family/friend influence and health/experience (this analysis on post test data only).
Rosi, 2017, Italy [23]	Intervention 1: baseline and during intervention (24 weeks). Intervention 2: during intervention (24 weeks	Machine sales/24 weeks period; % healthy/unhealthy items sold	Intervention 1—Sales: No significant change. Proportion of healthy/unhealthy items sold: significant change in favor of healthy items (ratio 3:97 to 35:65, *p* < 0.001). Intervention 2—Sales: No significant differences between groups. Proportion of healthy/unhealthy items sold: No significant between-group differences overall proportions. Proportion of least healthy items sold significantly lower in intervention 2b vs. 1 (19% vs. 28%, *p* < 0.05).
Seah, 2018, Singapore [24]	During intervention: 9 weeks data collection (3 weeks per intervention sequence)	Average weekly units of high-/lower-sugar beverages sold	Units sold: No significant differences between groups (% high-sugar beverages sold/week, control: 54%, tax messages: 53%, subsidy messages: 54%).
Tsai, 2018, Australia [27]	2017, 2018 (audit conducted once during each year)	Adherence to the New South Wales Healthy Food and Drink for Health Facilities Framework	Proportion of core to discretionary items changed from 23%/77% to 77%/23%. Proportion of SSBs changed from 56% of beverages to 0%. i.e., meeting criteria of the framework
Viana, 2018, USA [14]	Baseline and during for machine sales data (2 months data collection in 2012/2013)/During intervention for customer survey (Oct–Nov 2013)	Monthly machine sales data: (1) Revenue, (2) Profit, and (3) % of healthier products sold Point-of-purchase customer survey: Intended purchase item/reason	Revenue: No significant differences between or within groups. Profits: significantly increased in intervention machines. Proportion of healthier products purchased: significantly higher from intervention machines than controls (21.3% vs. 1.3%, *p* < 0.001). Purchase intention: 63% of customers had no purchase intention. Of these, customers at intervention machines were more likely to purchase healthier items than at control machines (50% vs. 10%, *p* < 0.01).

**Table 3:** Nutrition interventions in vending machines in the university setting: Risk of bias of included studies.

First Author, Year	Criteria										Overall Rating
	1	2	3	4	5	6	7	8	9	10	
Bergen, 2006 [17]	Y	Y	Y	NA	NA	Y	Y	Y	Y	Y	Positive
Brown, 2014 [18]	Y	Y	NA	NA	NA	Y	Y	Y	Y	Y	Positive
Dingman, 2015 [19]	Y	Y	Y	Y	NA	Y	Y	Y	Y	Y	Positive
French, 1997 [20]	Y	U	NA	NA	NA	Y	U	Y	Y	U	Neutral
Hoerr, 1993 [25]	Y	Y	NA	NA	NA	Y	Y	Y	Y	Y	Positive
Hua, 2017 [13]	Y	Y	Y	Y	NA	Y	Y	Y	Y	Y	Positive
Lapp, 2014 [26]	Y	Y	NA	Y	NA	Y	U	Y	Y	U	Neutral
Larson-Brown, 1978 [21]	Y	U	NA	NA	NA	Y	Y	Y	Y	Y	Neutral
Rose, 2018 [22]	Y	Y	NA	Y	NA	Y	U	Y	Y	Y	Neutral
Rosi, 2017 [23]	Y	Y	Y	NA	NA	Y	Y	Y	Y	Y	Positive
Seah 2018 [24]	Y	Y	Y	NA	NA	Y	Y	Y	Y	N	Positive
Tsai, 2018 [27]	Y	U	NA	NA	NA	Y	Y	Y	Y	U	Neutral
Viana, 2017 [14]	Y	Y	U	Y	NA	Y	Y	Y	Y	Y	Neutral

Criteria assess (1) clarity of the research question, (2) bias in the selection of study participants/subjects, (3) comparability of study groups, (4) whether methods of handling withdrawals were described, (5) the use of blinding, (6) whether intervention and comparators were described in detail, (7) whether outcomes were defined clearly and measurements were valid and reliable, (8) appropriateness of statistical analyses, (9) whether conclusions are supported by results and consider biases and limitations, and (10) whether study funding or conflicts of interest are likely to have introduced bias. Y: Yes; NA; Not Applicable; U: Unclear; N: No.

## Supplementary Materials

The following are available online at https://www.mdpi.com/2072-6643/12/3/876/s1, Table S1: Ovid Medline search strategy to identify nutrition interventions in vending machines in the University setting.

## References

[1] Roy R., Hebden L., Kelly B., De Gois T., Ferrone E.M., Samrout M., Vermont S., Allman-Farinelli M. (2016). Description, measurement and evaluation of tertiary-education food environments. Br. J. Nutr..

[2] Horacek T.M., Erdman M.B., Byrd-Bredbenner C., Carey G., Colby S.M., Greene G.W., Guo W., Kattelmann K.K., Olfert M., Walsh J. (2013). Assessment of the dining environment on and near the campuses of fifteen post-secondary institutions. Public Health Nutr..

[3] National Center for Education Statistics Digest of Education Statistics: 2017. https://nces.ed.gov/programs/digest/d17/.

[4] Universities Australia Data Snapshot. https://www.universitiesaustralia.edu.au/australias-universities/key-facts-and-data#.XJAbB6I6y6R.

[5] Byrd-Bredbenner C., Johnson M., Quick V.M., Walsh J., Greene G.W., Hoerr S., Colby S.M., Kattelmann K.K., Phillips B.W., Kidd T. (2012). Sweet and salty. An assessment of the snacks and beverages sold in vending machines on US post-secondary institution campuses. Appetite.

[6] Roy R., Rangan A., Hebden L., Yu Louie J.C., Tang L.M., Kay J., Allman-Farinelli M. (2017). Dietary contribution of foods and beverages sold within a university campus and its effect on diet quality of young adults. Nutrition.

[7] Okanagan Charter: An International Charter for Health Promoting Universities and Colleges. Proceedings of the 2015 International Conference on Health Promoting Universities and Colleges VII International Congress.

[8] Park H., Papadaki A. (2016). Nutritional value of foods sold in vending machines in a UK University: Formative, cross-sectional research to inform an environmental intervention. Appetite.

[9] Grech A., Hebden L., Roy R., Allman-Farinelli M. (2017). Are products sold in university vending machines nutritionally poor? A food environment audit. Nutr. Diet..

[10] Horacek M.T., Yildirim D.E., Matthews Schreiber M., Byrd-Bredbenner C., Colby S., White A.A., Shelnutt P.K., Olfert D.M., Mathews E.A., Riggsbee K. (2019). Development and validation of the vending evaluation for nutrient-density (vend)ing audit. Int. J. Environ. Res. Public Health.

[11] Roy R., Kelly B., Rangan A., Allman-Farinelli M. (2015). Food environment interventions to improve the dietary behavior of young adults in tertiary education settings: A systematic literature review. J. Acad. Nutr. Diet..

[12] Grech A., Allman-Farinelli M. (2015). A systematic literature review of nutrition interventions in vending machines that encourage consumers to make healthier choices. Obes Rev..

[13] Hua S.V., Kimmel L., Van Emmenes M., Taherian R., Remer G., Millman A., Ickovics J.R. (2017). Health Promotion and Healthier Products Increase Vending Purchases: A Randomized Factorial Trial. J. Acad. Nutr. Diet..

[14] Viana J., Leonard S.A., Kitay B., Ansel D., Angelis P., Slusser W. (2018). Healthier vending machines in a university setting: Effective and financially sustainable. Appetite.

[15] Moher D., Shamseer L., Clarke M., Ghersi D., Liberati A., Petticrew M., Shekelle P., Stewart L.A. (2015). Preferred reporting items for systematic review and meta-analysis protocols (PRISMA-P) 2015 statement. Syst. Rev..

[16] Academy of Nutrition and Dietetics (2012). Evidence Analysis Manual: Steps in the Academy Evidence Analysis Process.

[17] Bergen D., Yeh M.C. (2006). Effects of energy-content labels and motivational posters on sales of sugar-sweetened beverages: Stimulating sales of diet drinks among adults study. J. Am. Diet. Assoc..

[18] Brown M.V., Flint M., Fuqua J. (2014). The effects of a nutrition education intervention on vending machine sales on a university campus. J. Am. Coll. Health.

[19] Dingman D.A., Schulz M.R., Wyrick D.L., Bibeau D.L., Gupta S.N. (2015). Does providing nutrition information at vending machines reduce calories per item sold?. J. Public Health Policy.

[20] French S.A., Jeffery R.W., Story M., Hannan P., Snyder M.P. (1997). A pricing strategy to promote low-fat snack choices through vending machines. Am. J. Public Health.

[21] Larson-Brown L.B. (1978). Point-of-purchase information on vended foods. J. Nutr. Educ..

[22] Rose A.M., Williams R.A., Hanks A.S., Kennel J.A., Gunther C. (2018). Milk Vending Does Not Improve College Students’ Milk and Calcium Intakes. Health Promot. Pract..

[23] Rosi A., Zerbini C., Pellegrini N., Scazzina F., Brighenti F., Lugli G. (2017). How to improve food choices through vending machines: The importance of healthy food availability and consumers’ awareness. Food Qual. Prefer..

[24] Seah S.S.Y., Rebello S.A., Tai B.C., Tay Z., Finkelstein E.A., van Dam R.M. (2018). Impact of tax and subsidy framed messages on high- and lower-sugar beverages sold in vending machines: A randomized crossover trial. Int. J. Behav. Nutr. Phys. Act..

[25] Hoerr S.M., Louden V.A. (1993). Can nutrition information increase sales of healthful vended snacks?. J. Sch. Health.

[26] Lapp J.L., Ressler W.H., Frith A.L. (2014). College students, vending machines, and improving nutritional choices: The effects of adding healthier foods on perceptions of vending machines. Int. J. Food Saf. Nutr. Public Health.

[27] Tsai C., Slater S., Ronto R., Gebel K., Wu J.H.Y. (2018). Removal of sugary drinks from vending machines: An Australian university case study. Aust. N. Z. J. Public Health.

[28] Dingman D.A. (2013). Food Away from Home: Predicting Frequency and Changing Selections. Ph.D. Thesis.

[29] U.S. Food and Drug Administration Vending Machine Labeling Requirements. https://www.fda.gov/food/food-labeling-nutrition/vending-machine-labeling-requirements.

[30] Victoria State Government Healthy Choices Framework. http://heas.health.vic.gov.au/sites/default/files/HEAS-healthy-choices-framework.pdf.

[31] Matthews M.A., Horacek T.M. (2015). Vending machine assessment methodology. A systematic review. Appetite.

